# A Photonics-Assisted Binary/Quaternary Phase-Coded Microwave Signal Generator Applicable to Digital I/O Interfaces

**DOI:** 10.3390/mi14051034

**Published:** 2023-05-11

**Authors:** Jing Yin, Yan Zhao, Feng Yang, Dengcai Yang, Xiaoyu Wang

**Affiliations:** 1 Institute of Laser Engineering, Faculty of Materials and Manufacturing, Beijing University of Technology, Beijing 100124, China; 2 Department of Physics and Optoelectronic Engineering, Faculty of Science, Beijing University of Technology, Beijing 100124, China; 3 Beijing Engineering Research Center of Precision Measurement Technology and Instruments, Beijing University of Technology, Beijing 100124, China

**Keywords:** reconfigurable carrier frequency, phase-coded signal, signal generation, microwave photonics

## Abstract

A photonics-assisted binary/quaternary phase-coded microwave signal generator with fundamental/doubling reconfigurable carrier frequency applicable to digital I/O interfaces is proposed and has been verified by experiments. This scheme is based on a cascade modulation scheme, which is used to reconfigure fundamental/doubling carrier frequency and load the phase-coded signal, respectively. By controlling the radio frequency (RF) switch and the bias voltages of the modulator, the switching of the fundamental or doubling carrier frequency can be realized. When the amplitudes and sequence pattern of the two independent coding signals are set reasonably, binary or quaternary phase-coded signals can be realized. The sequence pattern of coding signals is applicable to digital I/O interfaces and can be directly generated through the IO interfaces of FPGA instead of an expensive high-speed arbitrary waveform generator (AWG) or other digital-to-analog conversion (DAC) systems. A proof-of-concept experiment is carried out, and the performance of the proposed system is evaluated from the aspects of phase recovery accuracy and pulse compression capability. In addition, the influence of residual carrier suppression and polarization crosstalk in non-ideal states on phase shifting based on polarization adjustment has also been analyzed.

## 1. Introduction

In modern radar systems, pulse compression technology can improve the time-bandwidth product (TBWP), which has a good advantage in solving the contradiction between the detection range and range resolution. The phase-coded microwave signal is a typical waveform of the pulse compression signal. However, due to the frequency dependence of electronic devices and the limited bandwidth of the system, it cannot meet the high frequency and broadband requirements. Microwave photonic technology is considered an effective way to overcome the limitations of electronic methods because it can generate high-frequency broadband and tunability phase-coded microwave signals with its advantages, such as a wide bandwidth, low transmission loss, and strong anti-electromagnetic interference ability [1,2,3,4].

To date, many schemes based on photonics have been proposed to realize phase-coded signals, for example, schemes based on spectral shaping and frequency-time mapping [5,6,7,8]. These methods can generate arbitrary waveforms through computer programming and are flexible and reconfigurable, but the system is complex and easily causes large power losses. The other scheme is based on an optical heterodyne, which has been widely studied because of its simple structure, high compactness, and stability. The difference in the phase modulation efficiency of dual polarization light signals in cascaded polarization modulators (PolM) or phase modulators (PM) is used to realize phase coding. Phase-coded signals with fundamental/doubling frequency and different phases can be generated by two cascaded PolMs [9], a dual-parallel PolM (DP-PolM) cascaded with PolM [10] and a dual-polarization dual-parallel Mach–Zehnder modulator (DP-DPMZM) cascaded with a PM [11] or PolM [12]. Fiber Bragg gratings (FBGs) were used to separate the two modulated optical sidebands [13,14] and then a PM was used to load the phase-coded signal in one of the sidebands, or the optical carrier was directly divided into two parts, one of which loads the local oscillator (LO) signal through an optical frequency shifter and the other carries out phase modulation with a PM [15]. Finally, those two signals are combined to beat and obtain the phase-coded signal. In addition, the phase-coded signal may also be generated based on a single parallel structure integrated modulator with multiple radio frequency (RF) ports, in which the upper and lower branches are loaded with microwave carrier frequency and a coding signal, respectively. A dual-drive Mach–Zehnder modulator (DD-MZM) [16], dual-parallel dual-drive Mach–Zehnder modulator (DP-DDMZM) [17], dual-polarization DD-MZM (DP-DDMZM) [18], or DP-DPMZM [19,20] was used to generate phase-coded signals.

Since multiphase-coded signals have better Doppler tolerance and a higher spectrum utilization ratio, it is of great significance to generate multiphase-coded signals. In [12,15,16,19,21], binary or quaternary phase-coded signals are realized by changing the waveform of the coding signals (square or multilevel wave). To generate a high-speed multilevel voltage waveform, a high-speed arbitrary waveform generator (AWG) or digital-to-analog conversion (DAC) should be needed. Two independent coding electrical signals can be used to drive one arm of DP-DPMZM [20] or DP-DDMZM [18], which can generate quadrature phase shift key (QPSK) signals or quaternary phase-coded signals. However, the frequencies of the two systems are limited since they cannot operate in frequency multiplying mode, and the reconfiguration flexibility of the system is low.

To fill in the above gaps, a photonics-assisted binary/quaternary phase-coded microwave signal generator with fundamental/doubling reconfigurable carrier frequency applicable to digital I/O interfaces is proposed and experimentally demonstrated. It is based on the cascade electro-optical modulators scheme, and the DP-DPMZM and polarization-dependent cascade phase modulator (PDC-PM) are used to reconfigure the fundamental/doubling carrier frequency and load the phase-coded signal, respectively. The proposed scheme has the following advantages. Firstly, multiple functions, including switchable fundamental/doubling carrier frequency and multiphase coding, are compacted in a cascade microwave photonic link (MPL). By controlling the RF switch and the bias voltages of the modulator, switching of the fundamental or doubling carrier frequency can be realized. When the amplitudes and sequence patterns of the two independent two-level coding signals are set reasonably, binary or quaternary phase-coded signals can be realized. Secondly, by using a specially designed integrated PDC-PM, a scheme applicable to loading two-level coding signals generated by digital I/O interfaces (such as LVCOMS, LVTTL, LVDS, and so on) is proposed. When generating binary phase-coded signals, the performance requirements for electric amplifiers can be effectively reduced. When generating quaternary phase-coded signals, they can be obtained by using two-level coding signals directly generated by the IO interface of FPGA instead of expensive high-speed AWG or other DAC systems. Thirdly, phase shifting is realized by adjusting the driving voltage of the PDC-PM, which allows the phase of the coded signal to be independently and continuously tuned to obtain a high-precision phase-coded signal. The performance of the proposed system is evaluated from the aspects of phase recovery accuracy and pulse compression performance of binary/quaternary phase-coded signals with reconfigurable carrier frequencies. The proposed scheme features a compact configuration and large operational frequency range, which can be employed in high-frequency and wideband radar systems.

## 2. Principle

**Figure 1:**
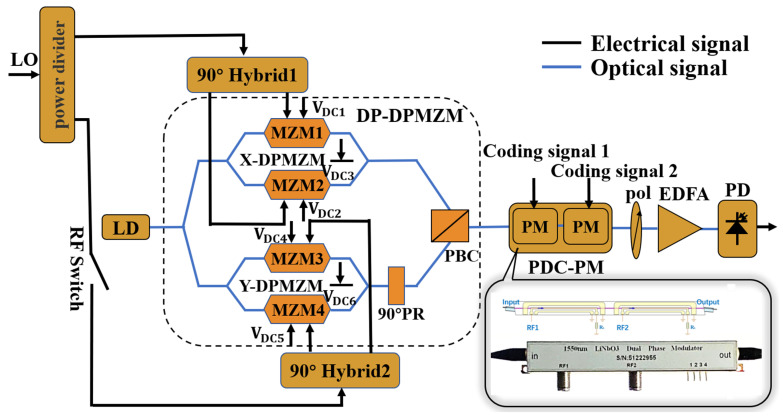
Schematic diagram of the binary and quaternary phase-coded microwave signals with fundamental/doubling frequencies. LD: laser diode. DP-DPMZM: dual-polarization dual-parallel Mach–Zehnder modulator. PR: polarization rotator. PBC: polarization beam combiner. PDC-PM: polarization-dependent cascade phase modulator. Pol: polarizer. EDFA: erbium-doped fiber amplifier. PD: photodetector. LO: local oscillator signal. RF Switch: radio frequency switch.

Figure 1 shows a schematic of the proposed photonics-assisted binary/quaternary phase-coded microwave signal generator with fundamental/doubling reconfigurable carrier frequency applicable to digital I/O interfaces. It contains a laser diode (LD), a power divider, an RF switch, a DP-DPMZM, two 90° hybrid couplers (HC), a PDC-PM, a polarizer (Pol), an erbium-doped optical amplifier (EDFA), and a photodetector (PD). The DP-DPMZM is composed of two sub-DPMZMs (X-DPMZM and Y-DPMZM) in parallel arms with a 90° polarization rotator (PR) after the Y-DPMZM. A polarization beam combiner (PBC) combines the output of the two sub-DPMZMs. Each sub-DPMZM consists of two sub-MZMs and three DC bias voltages, which are named sub-MZMn (n=1,2…4) and $V_{DCi}$ (i=1,2…6), respectively.

A continuous light wave produced by the LD is injected into the DP-DPMZM. The LO signal is divided into two parts through a power divider, one of which is input into the X-DPMZM through a 90° HC. When the DC bias voltage of the X-DPMZM is set reasonably, the +1st-order carrier-suppressed single-sideband (CS-SSB) modulation of the LO signal in the X-DPMZM can be obtained. The other part is controlled by an RF switch. When the RF switch is in open mode, the Y-DPMZM only outputs the optical carrier with a DC bias setting in the Y polarization state. When the RF switch is in closed mode, the LO signal is input into the Y-DPMZM through another 90° HC. By reasonably setting the DC bias voltage of the Y-DPMZM, the −1st-order CS-SSB of the LO signal in the Y polarization state can be reasonably obtained. Then, the polarization-multiplexed signal is input into the PDC-PM, which is driven by two independent coding signals. When the coding sequence mode is changed, a binary or quaternary phase-coded signal can be obtained after the PD, and the carrier frequency is equal to or twice that of the LO signal.

The optical carrier from the LD is given by $E_{in}(t)=E_{0}\exp(j\omega_{0}t)$, where $E_{0}$ and $\omega_{0}$ represent the amplitude and angular frequency of the optical carrier, respectively. The LO signal is divided into two parts by a power divider, one part of which is input into the X-DPMZM through a 90° HC. The optical signal at the output of the X-DPMZM is

(1) $$E_{X-DPMZM}(t)\approx \frac{\sqrt{2}}{8}E_{in}(t)\left\{\begin{array}{l} \left[\begin{array}{l} \exp(jm_{LO}\cos(\omega_{LO}t))\\ +\exp(-jm_{LO}\cos(\omega_{LO}t))\exp(j\varphi_{1}) \end{array}\right]\\ +\left[\begin{array}{l} \exp(jm_{LO}\cos(\omega_{LO}t+\pi /2))\\ +\exp(-jm_{LO}\cos(\omega_{LO}t+\pi /2))\exp(j\varphi_{2}) \end{array}\right]\exp(j\varphi_{3}) \end{array}\right\},$$

where $\omega_{LO}$ is the angular frequency of the LO signal, $m_{LO}=\pi V_{LO}/V_{\pi }$ is the modulation index of the sub-MZM, $\varphi_{i}=\pi V_{DCi}/V_{\pi }(i=1,2,3)$ corresponds to the phase shifts of the sub-MZM in the X-DPMZM induced by DC bias, and $V_{\pi }$ is the half-wave voltage of the DP-DPMZM.

By biasing the two sub-MZMs at the minimum transmission point (MITP) and the main MZM at the quadrature transmission point (QTP), which means $\varphi_{1}=\varphi_{2}=\pi$ and $\varphi_{3}=-\pi /2$, the +1st-order CS-SSB of the LO signal can be obtained. In the small-signal modulation approximation, the high-order modulations (*n* ≥ 2) are neglected. It can be given by

(2) $$E_{X-DPMZM}(t)\approx \frac{\sqrt{2}}{2}E_{0}J_{1}(m_{LO})\exp(j\left[(\omega_{0}+\omega_{LO})t+\frac{\pi }{2}\right]),$$

where $J_{n}(\cdot )$ is the nth-order Bessel function of the first kind.

### 2.1. Generation of Binary/Quaternary Phase-Coded Signals with Fundamental Frequency

When the RF switch is in the open state, the Y-DPMZM is only biased with DC voltage. Setting $\varphi_{4}=\varphi_{5}=\varphi_{6}=2\pi$, the output of the Y-DPMZM is given by

(3) $$E_{Y-DPMZM}(t)\approx \frac{\sqrt{2}}{2}E_{0}\exp(j\omega_{0}t),$$

where $\varphi_{4}$, $\varphi_{5}$, and $\varphi_{6}$ correspond to the phase shifts of the sub-MZM in the Y-DPMZM induced by DC bias. Therefore, the optical field of the DP-DPMZM can be expressed as

(4) $$E_{DP-DPMZM}(t)=\left[\begin{array}{l} E_{X-DPMZM}\cdot \vec{e_{X}}\\ E_{Y-DPMZM}\cdot \vec{e_{Y}} \end{array}\right]\approx \left[\begin{array}{c} \frac{\sqrt{2}}{2}E_{0}J_{1}(m_{LO})\exp(j\left[(\omega_{0}+\omega_{LO})t+\frac{\pi }{2}\right])\cdot \vec{e_{X}}\\ \frac{\sqrt{2}}{2}E_{0}\exp(j\omega_{0}t)\cdot \vec{e_{Y}} \end{array}\right],$$

Then, the polarization multiplexed optical signal is input into the PDC-PM. The PDC-PM has two independent RF ports, and its structure is shown in Figure 1. Since optical signals with different polarization states have different modulation efficiencies for the polarization-dependent phase modulator, the output of the PDC-PM can be expressed as

(5) $$E_{PDC-PM}(t)\approx \left\{\begin{array}{l} \frac{\sqrt{2}}{2}E_{0}J_{1}(m_{LO})\exp(j\left[(\omega_{0}+\omega_{LO})t+\frac{\pi }{2}\right])\exp(j\varphi_{1x}+j\varphi_{2x})\cdot \vec{e_{X}}\\ \frac{\sqrt{2}}{2}E_{0}\exp(j\omega_{0}t)\exp(j\varphi_{1y}+j\varphi_{2y})\cdot \vec{e_{Y}} \end{array}\right\},$$

where $\varphi_{nx}$ and $\varphi_{ny}$ (n=1,2) represent the phase shifts of the X and Y polarization state optical signals introduced by the two cascade sub-PMs of the PDC-PM, respectively. A Pol is used to project the orthogonally polarized optical signal to the same polarization state, and the output of the Pol can be given by

(6) $$E_{pol}(t)\approx \left\{\begin{array}{l} \frac{1}{2}E_{0}J_{1}(m_{LO})\exp(j\left[(\omega_{0}+\omega_{LO})t+\frac{\pi }{2}\right])\exp(j\varphi_{1x}+j\varphi_{2x})\\ +\frac{1}{2}E_{0}\exp(j\omega_{0}t)\exp(j\varphi_{1y}+j\varphi_{2y}) \end{array}\right\},$$

Suppose that the responsivity of the PD is ℜ. Ignoring the DC component, the photocurrent of the PD can be expressed as

(7) $$i_{fun}(t)=ℜ\cdot E_{pol}(t)\cdot E_{pol}^{*}(t)\approx \frac{1}{4}ℜE_{0}^{2}J_{1}(m_{LO})\cos\left[\omega_{LO}t+\frac{\pi }{2}+(\varphi_{1x}+\varphi_{2x}-\varphi_{1y}-\varphi_{2y})\right],$$

For the polarization-dependent phase modulator, the modulation efficiency in TM polarization is approximately three times that in TE polarization [22], and the optical phase difference is linearly related to the voltage of the PDC-PM (The specific structure and principle of PDC-PM can be seen in Appendix A). Hence,

(8) $$\varphi_{nx}-\varphi_{ny}=\frac{2\pi V_{n}\cdot b_{n}(t)}{3V_{\pi ,PM}},$$

where $V_{n}$ is the amplitude of the driving voltage and $V_{\pi ,PM}$ is the half-wave voltage of the PDC-PM for TM polarization.

Assume that the PDC-PM is driven by two independent coding signals, denoted by $S_{1}(t)=V_{1}\cdot b_{1}(t)$ and $S_{2}(t)=V_{2}\cdot b_{2}(t)$, respectively, and $b_{n}(t)$ is the coding pattern. Then, Equation (7) can be written as

(9) $$i_{fun}(t)\approx \frac{1}{4}ℜE_{0}^{2}J_{1}(m_{LO})\cos\left[\omega_{LO}t+\frac{\pi }{2}+\frac{2\pi V_{1}\cdot b_{1}(t)}{3V_{\pi ,PM}}+\frac{2\pi V_{2}\cdot b_{2}(t)}{3V_{\pi ,PM}}\right],$$

Equation (9) shows that by adjusting the amplitude of the coding signal loaded into the PDC-PM, an arbitrary phase-coded signal with fundamental carrier frequency can be realized. Both one port and two ports could be selected to load the coding signals in binary phase-coded mode. Loading the same signal into the two ports can reduce the requirements on the amplification amplitude of the electric amplifier. The amplitudes and coding sequence pattern rules for binary and quaternary phase-coded signals are shown in Table 1 and Table 2.

Selecting two ports to load the coding signals with the same amplitude in binary phase-coded mode, that is, $V_{1}=V_{2}=3V_{\pi ,PM}/4$, Equation (9) can be written as

(10) $$i_{fun\_B}(t)\approx \left\{\begin{array}{l} \frac{1}{4}ℜE_{0}^{2}J_{1}(m_{LO})\cos(\omega_{LO}t+\frac{\pi }{2})\;\;\;\;\;\;\;\;\;\;\;(b_{1}(t)=0,b_{2}(t)=0)\\ \frac{1}{4}ℜE_{0}^{2}J_{1}(m_{LO})\cos(\omega_{LO}t+\frac{3\pi }{2})\;\;\;\;\;\;\;\;\;\;(b_{1}(t)=1,b_{2}(t)=1) \end{array}\right.,$$

In quaternary phase-coded mode, i.e., $V_{1}=3V_{\pi ,PM}/2$ and $V_{2}=3V_{\pi ,PM}/4$, Equation (9) can be written as

(11) $$i_{fun\_Q}(t)\approx \left\{\begin{array}{l} \frac{1}{4}ℜE_{0}^{2}J_{1}(m_{LO})\cos(\omega_{LO}t+\frac{\pi }{2})\;\;\;\;\;\;\;\;\;\;\;(b_{1}(t)=0,b_{2}(t)=0)\\ \frac{1}{4}ℜE_{0}^{2}J_{1}(m_{LO})\cos(\omega_{LO}t+\pi )\;\;\;\;\;\;\;\;\;\;\;\;(b_{1}(t)=0,b_{2}(t)=1)\\ \frac{1}{4}ℜE_{0}^{2}J_{1}(m_{LO})\cos(\omega_{LO}t+\frac{3\pi }{2})\;\;\;\;\;\;\;\;\;\;(b_{1}(t)=1,b_{2}(t)=0)\\ \frac{1}{4}ℜE_{0}^{2}J_{1}(m_{LO})\cos(\omega_{LO}t+2\pi )\;\;\;\;\;\;\;\;\;\;(b_{1}(t)=1,b_{2}(t)=1) \end{array}\right.,$$

From Equations (10) and (11), binary or quaternary phase-coded signals with fundamental frequency are implemented.

### 2.2. Generation of Binary/Quaternary Phase-Coded Signals with Doubling Frequency

When the RF switch is in a closed state, the Y-DPMZM is driven by the LO signal through 90° HC. Setting $\varphi_{4}=\varphi_{5}=\pi$ and $\varphi_{6}=\pi /2$, the −1st-order CS-SSB of the LO signal can be obtained, and the output of the DP-DPMZM can be written as

(12) $$E_{DP-DPMZM}(t)=\left\{\begin{array}{l} \frac{\sqrt{2}}{2}E_{0}J_{1}(m_{LO})\exp(j[(\omega_{0}+\omega_{LO})t+\frac{\pi }{2}])\cdot \vec{e_{X}}\\ \frac{\sqrt{2}}{2}E_{0}J_{1}(m_{LO})\exp(j[(\omega_{0}-\omega_{LO})t+\frac{\pi }{2}])\cdot \vec{e_{Y}} \end{array}\right\},$$

Similarly, the PDC-PM is driven by two coding signals, and the output of the PD can be expressed as

(13) $$i_{dou}(t)\approx \frac{1}{2}ℜE_{0}^{2}J_{1}^{2}(m_{LO})\cos\left[2\omega_{LO}t-\frac{2\pi V_{1}\cdot b_{1}(t)}{3V_{\pi ,PM}}-\frac{2\pi V_{2}\cdot b_{2}(t)}{3V_{\pi ,PM}}\right],$$

Equation (13) shows that phase modulation signals with doubling carrier frequency are realized. By reasonably setting the amplitudes of two independent coding signals as shown in Table 1 and Table 2, a binary or quaternary phase-coded signal can be realized, and the phase-coded signal can be written as

(14) $$i_{dou\_B}(t)\approx \left\{\begin{array}{l} \frac{1}{2}ℜE_{0}^{2}J_{1}^{2}(m_{LO})\cos(2\omega_{LO}t)\;\;\;\;\;\;\;\;\;\;\;\;\;\;\;(b_{1}(t)=0,b_{2}(t)=0)\\ \frac{1}{2}ℜE_{0}^{2}J_{1}^{2}(m_{LO})\cos(2\omega_{LO}t-\pi )\;\;\;\;\;\;\;\;(b_{1}(t)=1,b_{2}(t)=1) \end{array}\right.,$$

(15) $$i_{dou\_Q}(t)\approx \left\{\begin{array}{l} \frac{1}{2}ℜE_{0}^{2}J_{1}^{2}(m_{LO})\cos(2\omega_{LO}t)\;\;\;\;\;\;\;\;\;\;\;\;\;\;\;(b_{1}(t)=0,b_{2}(t)=0)\\ \frac{1}{2}ℜE_{0}^{2}J_{1}^{2}(m_{LO})\cos(2\omega_{LO}t-\frac{\pi }{2})\;\;\;\;\;\;\;(b_{1}(t)=0,b_{2}(t)=1)\\ \frac{1}{2}ℜE_{0}^{2}J_{1}^{2}(m_{LO})\cos(2\omega_{LO}t-\pi )\;\;\;\;\;\;\;\;(b_{1}(t)=1,b_{2}(t)=0)\\ \frac{1}{2}ℜE_{0}^{2}J_{1}^{2}(m_{LO})\cos(2\omega_{LO}t-\frac{3\pi }{2})\;\;\;\;\;\;(b_{1}(t)=1,b_{2}(t)=1) \end{array}\right.,$$

Overall, by using the RF switch and setting the bias voltages of the Y-DPMZM, the optical carrier or −1st-order CS-SSB modulation of the LO signal can be obtained in the Y-polarization state. Then, combining it with the +1st-order CS-SSB modulation of the LO signal in the X-polarization state, the fundamental or doubling carrier frequency of the LO signal will be realized after photoelectric conversion. When the amplitudes and sequence patterns of the two independent coding signals are set reasonably, a binary or quaternary phase-coded signal can be realized. Due to the CS-SSB modulation of the LO signal in the proposed system both in fundamental and doubling carrier frequency modes, chromatic dispersion-induced power fading is effectively eliminated over the long-distance single-mode fiber [23].

In addition, the PDC-PM is driven by two independent two-level digital signals, which are only required to cooperate with the electrical amplifier with fixed gain and can generate the quaternary phase-coded signal, instead of using high-speed AWG or other DAC systems to generate multilevel coding signals, which can be directly generated through the IO interfaces of FPGA.

## 3. Results

**Figure 2:**
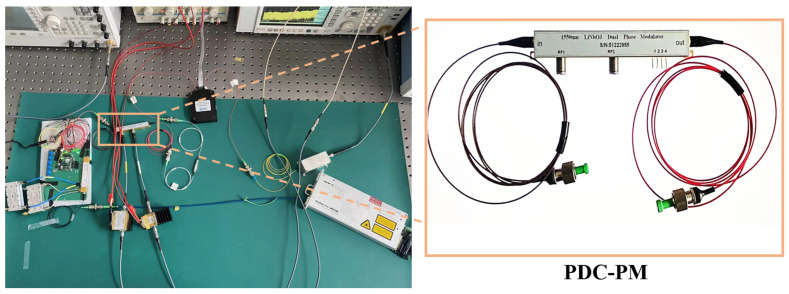
Experimental link diagram of the binary and quaternary phase-coded microwave signals with fundamental/doubling frequencies. The illustration is PDC-PM.

The experimental link was built according to the schematic diagram shown in Figure 2. An LD (NKT, Koheras BASIK X15, Beijing, China) is used to generate the optical carrier with a wavelength of 1550.21 nm and a power of 15.5 dBm, which is sent into the DP-DPMZM (Fujitsu, FTM7977HQA, Beijing, China) with a half-wave voltage of 3.5 V. The LO signal is generated by the microwave signal generator (MSG, Agilent, E8257D, Beijing, China) and sent to the DP-DPMZM through an electrical 90° hybrid coupler (Marki Microwave QH-0226, 2–26.5 GHz, Beijing, China). Limited by the equipment in our laboratory, we use an arbitrary waveform generator (Tektronix AWG70002B, Beijing, China) to take the place of FPGA, which only generates a two-level digital signal. Then, it is loaded into the PDC-PM after amplification with a gain-adjustable electric amplifier (Conquer, KG-EOD-10G, Beijing, China). A PD (Conquer, KG-PD-20G, 20 GHz, 0.75 A/W, Beijing, China) is used to realize photoelectric conversion. In the experiment, an EDFA is utilized to implement the loss of optical signals in the proposed link. An optical spectrum analyzer (OSA, Yokogawa, AQ6370C, Beijing, China) and an oscilloscope (Tektronix DPO75902SX, Beijing, China) are employed to monitor the optical spectra and waveforms, respectively.

### 3.1. Generation of Binary/Quaternary Phase-Coded Signals with Fundamental Frequency

**Figure 3:**
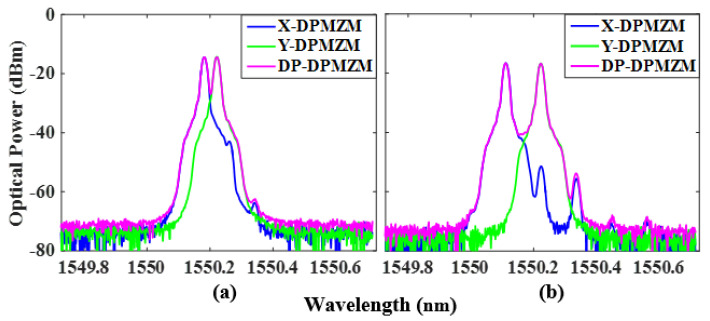
Measured output optical spectra of the X-DPMZM, Y-DPMZM, and DP-DPMZM with a frequency of (**a**) 5 GHz and (**b**) 14 GHz.

The phase-coded signal with fundamental frequency is demonstrated. LO signals of 5 GHz and 14 GHz are used to verify the performance of the proposed scheme with a fundamental frequency phase-coded signal. The power of the LO signal is 15 dBm, which is sent into the power divider and divided into two parts, one of which is input into the X-DPMZM through a 90° HC, and the DC bias voltage of the X-DPMZM satisfies $\varphi_{1}=\varphi_{2}=\pi$ and $\varphi_{3}=-\pi /2$. This allows the RF switch to be in the open state and sets the two sub-MZMs and the main MZM of the Y-DPMZM to work at the maximum transmission point (MATP). Then, the +1st-order CS-SSB modulation of the LO signal in the X- polarization state can be obtained. Since the output of the DP-DPMZM combines the X-DPMZM with the Y-DPMZM through a PBC and has an orthogonal polarization state, both can be distinguished by a polarization controller (PC) with a Pol. Figure 3a,b shows the optical spectra of the X-DPMZM, Y-DPMZM, and DP-DPMZM with frequencies of 5 GHz and 14 GHz. The carrier and −1st-order sideband suppression ratios in the X-DPMZM are 23.4 dB and 28.5 dB with a fundamental frequency of 5 GHz, and 34.7 dB and 38 dB with a fundamental frequency of 14 GHz, respectively.

**Figure 4:**
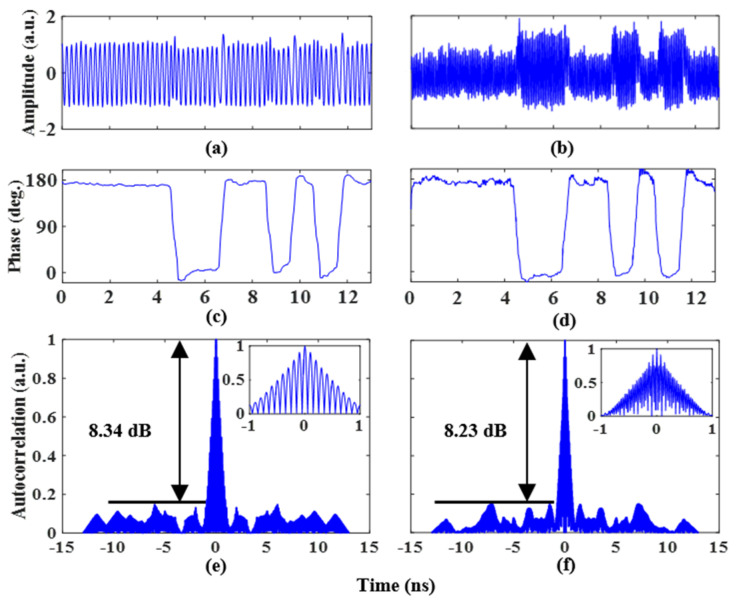
Binary phase-coded signal waveform with a frequency of (**a**) 5 GHz and (**b**) 14 GHz, (**c**) and (**d**) for the recovered phase information. Autocorrelations of the binary phase-coded signal at a frequency of (**e**) 5 GHz and (**f**) 14 GHz. The insets show the zoomed-in views of the corresponding autocorrelations.

Next, two coding signals ($S_{1}(t)$ and $S_{2}(t)$) generated by the AWG are amplified by an electrical amplifier and loaded into the two RF ports of the PDC-PM. Two 1 Gb/s coding signals with a length of 13 bits and a pattern of “1, 1, 1, 1, 1, 0, 0, 1, 1, 0, 1, 0, 1” are applied to the PDC-PM. To realize the binary phase-coded signal, the amplitude of the two coding signals is $3V_{\pi ,PM}/4$. Figure 4a,b shows the phase-coded time-domain waveforms of 5 GHz and 14 GHz microwave signals. The extracted phase information can be obtained with the Hilbert transform, and the results are shown in Figure 4c,d. The phase jump of π can be clearly observed from the experimental results, and its phase information fits well with the trend of the loaded coding signal, which is consistent with the theoretical analysis. To evaluate the pulse compression capability of the proposed system, the autocorrelations of the phase-coded signals are calculated. Figure 4e shows the phase-coded signal autocorrelation results at a frequency of 5 GHz, and the illustration shows an enlarged view. The peak-to-sidelobe ratio (PSR) and full width at half maximum (FWHM) are 8.34 dB and 0.83 ns, respectively. The pulse compression ratio (PCR) is calculated to be 15.6. The autocorrelation results at a frequency of 14 GHz are shown in Figure 4f. The PSR and FWHM are 8.23 dB and 0.72 ns, respectively. The PCR is calculated to be 18.

**Figure 5:**
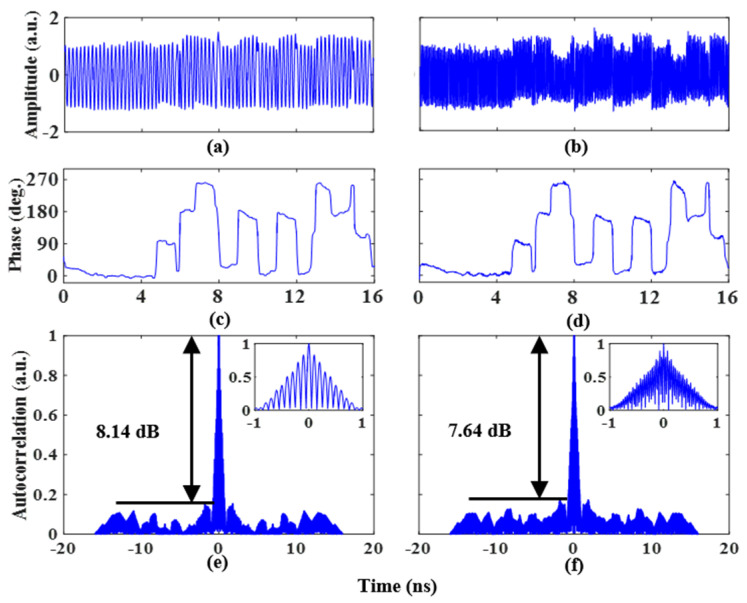
Quaternary phase-coded signal waveform with a frequency of (**a**) 5 GHz and (**b**) 14 GHz, (**c**,**d**) for the recovered phase information. Autocorrelations of the quaternary phase-coded signal at a frequency of (**e**) 5 GHz and (**f**) 14 GHz. The insets show the zoomed-in views of the corresponding autocorrelations.

The performance of the proposed scheme with the generation of quaternary phase-coded signals is also investigated. Two 1 Gb/s coding signals with a length of 16 bits and patterns of “0, 0, 0, 0, 0, 0, 1, 1, 0, 1, 0, 1, 0, 1, 1, 0” and “0, 0, 0, 0, 0, 1, 0, 1, 0, 0, 0, 0, 0, 1, 0, 1” are applied to the two RF ports of the PDC-PM, respectively. To realize the quaternary phase-coded signal, the amplitudes of the two coding signals need to comply with the rules described in Table 2. Therefore, the amplitudes of the coding signals obtained by adjusting the gain of the amplifier that is loaded to the two RF ports of the PDC-PM after amplification by the electric amplifier are $3V_{\pi ,PM}/2$ and $3V_{\pi ,PM}/4$, respectively. Figure 5a,b illustrates the waveforms of quaternary phase-coded microwave signals with frequencies of 5 GHz and 14 GHz, respectively. The extracted phase information can be obtained, and the results are shown in Figure 5c,d. The extracted phase shift has four levels, and the phase difference between each level is π/2. The pulse compression capability is studied by calculating the autocorrelation of the phase-coded signals. Figure 5e shows the phase-coded signal autocorrelation results at a frequency of 5 GHz. The PSR, FWHM, and PCR are 8.14 dB, 0.81 ns, and 19.7, respectively. The autocorrelation result at a frequency of 14 GHz is shown in Figure 5f. The PSR, FWHM, and PCR are 7.64 dB, 0.72 ns, and 22.2, respectively.

### 3.2. Generation of Binary/Quaternary Phase-Coded Signals with Doubling Frequency

**Figure 6:**
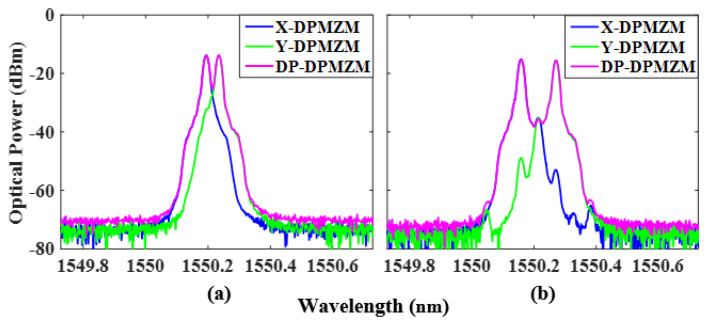
Measured output optical spectra of the X-DPMZM, Y-DPMZM, and DP-DPMZM with a frequency of (**a**) 2.5 GHz and (**b**) 7 GHz.

When the RF switch is closed, LO signals with frequencies of 2.5 GHz and 7 GHz are used to generate doubling carrier frequency phase-coded signals with carrier frequencies of 5 GHz and 14 GHz, respectively. Keeping the +1st-order CS-SSB modulation of the LO signal in the X-DPMZM and adjusting the DC bias voltage of the Y-DPMZM to satisfy $\varphi_{4}=\varphi_{5}=\pi$ and $\varphi_{6}=\pi /2$, the −1st-order CS-SSB of the LO signal can be obtained in the Y-polarization state. An OSA was used to observe the output optical spectra of the X- and Y-DPMZM and DP-DPMZM, and the spectra with a frequency of 2.5 GHz and 7 GHZ are shown in Figure 6a,b.

**Figure 7:**
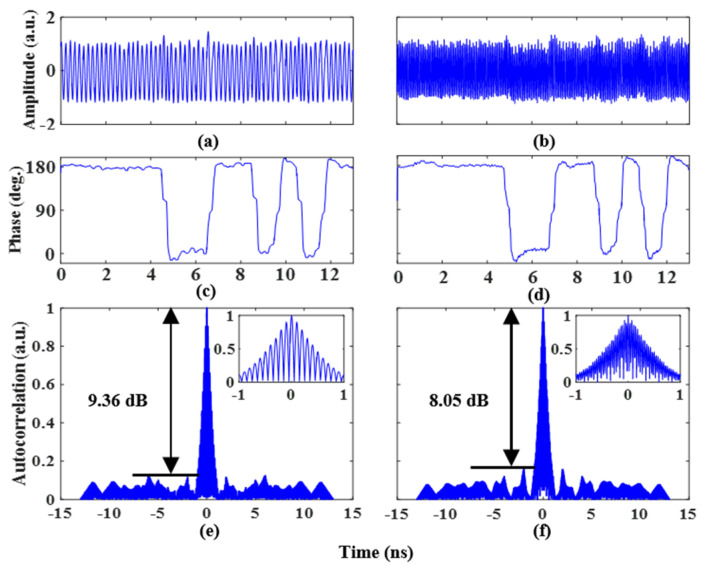
Binary phase-coded signal waveform with a doubling carrier frequency at a frequency of (**a**) 2.5 GHz and (**b**) 7 GHz, (**c**) and (**d**) for the recovered phase information. Autocorrelations of the binary phase-coded signal with a doubling carrier frequency at a frequency of (**e**) 2.5 GHz and (**f**) 7 GHz. The insets show the zoomed-in views of the corresponding autocorrelations.

The ability of the phase-coded signal with doubling carrier frequency is also proven. Two 1 Gb/s coding signals with a length of 13 bits and a pattern of “1, 1, 1, 1, 1, 0, 0, 1, 1, 0, 1, 0, 1” are applied to the two RF ports of the PDC-PM. Similarly, to realize the binary phase-coded signal, the amplitude of the coding signals is $3V_{\pi ,PM}/4$ after being amplified by an electric amplifier. Figure 7a,b illustrates the phase-coded waveform of the LO signals at 2.5 GHz and 7 GHz, respectively. The extracted phase information is shown in Figure 7c,d. The phase jump of π can also be clearly observed. Then, the pulse compression ability was confirmed. Figure 7e shows the phase-coded signal autocorrelation results at a frequency of 2.5 GHz. The PSR, FWHM, and PCR are 9.36 dB, 0.82 ns, and 15.8, respectively. The autocorrelation result at a frequency of 7 GHz is shown in Figure 7f. The PSR, FWHM, and PCR are 8.05 dB, 0.86 ns, and 15.1, respectively.

**Figure 8:**
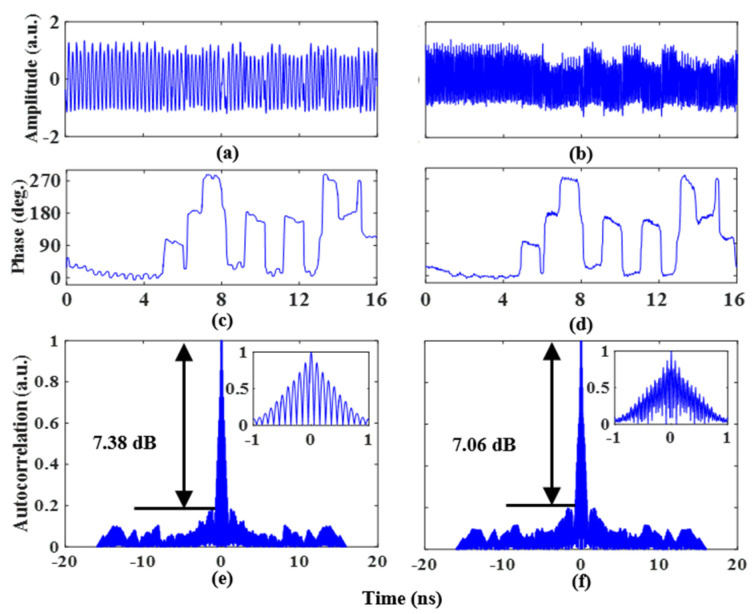
Quaternary phase-coded signal waveform with an LO frequency of (**a**) 2.5 GHz and (**b**) 7 GHz, (**c**,**d**) for the recovered phase information. Autocorrelations of the quaternary phase-coded signal with doubling carrier frequency at a frequency of (**e**) 2.5 GHz and (**f**) 7 GHz. The insets show the zoomed-in views of the corresponding autocorrelations.

To realize quaternary phase-coded signals with a doubling carrier frequency, the amplitudes of the two 1 Gb/s coding signals that are input into the PDC-PM are $3V_{\pi ,PM}/2$ and $3V_{\pi ,PM}/4$ after being amplified by the electric amplifier, and their coding patterns are “0, 0, 0, 0, 0, 0, 1, 1, 0, 1, 0, 1, 0, 1, 1, 0” and “0, 0, 0, 0, 0, 1, 0, 1, 0, 0, 0, 0, 0, 1, 0, 1”, respectively. Figure 8a,b illustrates the waveforms of the doubling carrier frequency quaternary phase-coded signals with 2.5 GHz and 7 GHz phase-coded microwave signals, respectively. The extracted phase information is shown in Figure 8c,d, and its phase information fits well with the trend of the loaded coding signal. Figure 8e shows the autocorrelation result of the quaternary phase-coded signal with doubling carrier frequency at a frequency of 2.5 GHz. The PSR, FWHM, and PCR are 7.38 dB, 0.82 ns, and 19.5, respectively. At a frequency of 7 GHz, the PSR, FWHM, and PCR are 7.06 dB, 0.86 ns, and 18.6, respectively, as shown in Figure 8f.

In the above, the LO signal with frequencies of 5 GHz and 14 GHz in the fundamental frequency mode and frequencies of 2.5 GHz and 7 GHz in the doubling frequency mode is used to verify the phase-coded signal generation of the proposed system at different LO frequencies. By loading a binary or quaternary coding signal, phase-coded signals with carrier frequencies of 5 GHz and 14 GHz were generated in both modes mentioned above. This indicates that the carrier frequency can be tuned in both the fundamental and doubling modes. In the proposed scheme, the working frequency is also limited by the bandwidth of the frequency-related devices, such as the RF switch, 90° hybrid coupler, DP-DPMZM modulator, and PD. However, the bandwidths of the commercial RF switches, 90° hybrid couplers, and PDs can reach up to 50 GHz, 40 GHz, and 100 GHz, respectively. The bandwidth of commercial DP-DPMZM modulators can also reach 32 GHz, so the carrier frequency can exceed 32 GHz in fundamental mode. In addition, in the doubling frequency mode, the RF switch, 90° hybrid coupler, and DP-DPMZM modulator only account for half of the system bandwidth. Therefore, the frequency adjustable range of this scheme is twice the modulation bandwidth of the DP-DPMZM in doubling frequency mode, and it can be greater than 60 GHz. The reconfigurable function of the carrier frequency in this scheme not only makes the system have a higher working frequency band, but also reduces the frequency requirement for the LO signal. In addition, the better carrier and sideband suppression ratio of the optical signal at the output of the DP-DPMZM can be achieved by optimizing the power of the LO signal within a certain range, which can reduce the impact of the optical carrier on the accuracy of phase shifting and spurious signal of the system. In theory, with a certain input power of the PD, the generated microwave signal is maximum when the carrier and the sideband have the same power [19]. In this scheme, the DP-DPMZM operates at normal points in both the fundamental and doubling frequency modes; thus, commercial bias controllers can be used to achieve stable operation.

Then, in the pulse compression radar system, theoretically, the PCR is equal to the coding length used. If a longer coding sequence is used, a larger PCR will be obtained. The rate of the coding signal is limited by the bandwidth of the device. If a higher coding rate is used, a narrower FWHM will be obtained after pulse compression, which can further improve the range resolution of the radar system. On the other hand, the autocorrelation function of the phase-coded signal mainly depends on the autocorrelation function of the coding signal used, while Barker code and Frank code are kinds of symbols with good autocorrelation.

Compared with other phase-coded microwave signal generator schemes in the references, the comparison of the proposed scheme in this manuscript in terms of link structure, reconfigurable characteristics, control complexity, and suitability for on-chip integration is shown in Table 3.

## 4. Discussion

### 4.1. Influence of the Carrier Residual

The above principal analysis is based on the ideal carrier suppression state, that is, both optical carriers at the output of the X- or Y-DPMZM are completely suppressed. Therefore, when the coding voltage of the PDC-PM is adjusted, only the phase of the microwave signal changes while the amplitude remains unchanged. However, due to the bias control accuracy and drift of the modulator, only a limited extinction ratio can be obtained, which makes it impossible to completely suppress the optical carrier. Therefore, the residual optical carriers will have some fluctuations in the amplitude of the coded signal, as shown in Figure 4b and Figure 5b. The influence of the carrier suppression ratio on the generation of the phase-coded signal is analyzed as follows.

In phase-coded signals with fundamental frequency mode, if the output of the X-DPMZM contains a residual optical carrier, Equation (2) should be expressed as

(16) $$E_{X-DPMZM}(t)\approx A\exp(j\left[(\omega_{0}+\omega_{LO})t+\frac{\pi }{2}\right])+B\exp(j\omega_{0}t),$$

where *A* and *B* represent the amplitudes of the +1st-order sideband and residual optical carrier, respectively. The difference between *A* and *B* (in dBm) is the carrier suppression ratio of the X-DPMZM modulator. Meanwhile, Equation (3) should also be rewritten as

(17) $$E_{Y-DPMZM}(t)\approx C\exp\left(j\omega_{0}t\right),$$

where *C* represents the amplitude of the Y- polarization optical carrier. Therefore, the polarization multiplexed signal modulated by the coding signals after the PDC-PM should be written as

(18) $$E_{PDC-PM}(t)\approx \left\{\begin{array}{l} \left[A\exp\left[j(\omega_{0}+\omega_{LO})t+\pi /2\right]+B\exp(j\omega_{0}t)\right]\exp(j\varphi_{1x}+j\varphi_{2x})\cdot \vec{e_{X}}\\ C\exp(j\omega_{0}t)\exp(j\varphi_{1y}+j\varphi_{2y})\cdot \vec{e_{Y}} \end{array}\right\},$$

The signals are injected into Pol at 45°, and then the electrical current after the PD can be expressed as

(19) $$i(t)\approx AB\cos(\omega_{LO}t+\pi /2)+AC\cos(\omega_{LO}t+\pi /2+\psi ),$$

where $\psi =\varphi_{1x}+\varphi_{2x}-\varphi_{1y}-\varphi_{2y}$. The signal contains not only the fundamental frequency phase-coded signal, but also a term with the same frequency and fixed phase. After simplification, Equation (19) can be written as

(20) $$i(t)\approx K\cos(\omega_{LO}t+\theta ),$$

where $\theta =\arcsin\left\{\left[B+C\cos\left(\psi \right)\right]/\sqrt{B^{2}+2BC\cos\psi +C^{2}}\right\}$ and $K=A\sqrt{B^{2}+2BC\cos\psi +C^{2}}$ are the phase and amplitude of vector synthesis, respectively, and both are related to carrier residue *B*.

**Figure 9:**
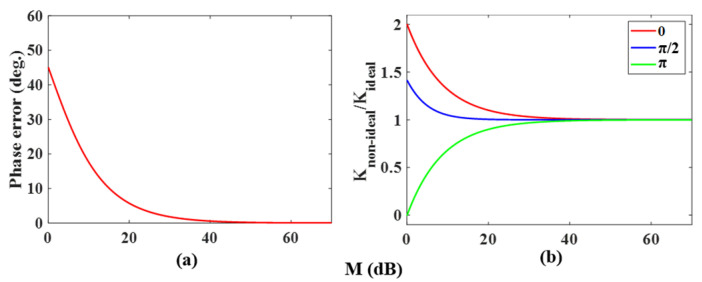
(**a**) Relationship between the phase changes of the output signal and carrier suppression ratio when the PDC-PM driving voltage corresponds to π/2; (**b**) the variation in the microwave signal amplitude ratio between the nonideal state and ideal state with the carrier suppression ratio at different phase shift settings.

To observe the influence of the carrier residue on the phase and amplitude of the coding signal, let M=A−B represent the carrier suppression ratio in the X-polarization state. In phase coding, three phases, 0, π/2 and π, are mainly used. When the PDC-PM is only driven by DC voltages, setting the driving voltage on the PDC-PM corresponds to a phase shifting of 0. From Equation (19), it can be obtained that the phase of the vector synthesis signal of the fixed phase term and the modulated phase term is always 0. When the driving voltage on PDC-PM corresponds to the phase shifting of π, the phase of the vector synthesis signal is 0 or π, depending on their amplitude difference and related to the carrier suppression ratio M. When the driving voltage on PDC-PM corresponds to the phase shifting of π/2, the change in the synthesis phase with the carrier suppression ratio is shown in Figure 9a, where the phase in the ideal state of the carrier residue is used as the reference. With the increase in the carrier suppression ratio *M*, the phase tends to 0, that is, it approaches the ideal state. For different phase shifting, the variation in the microwave signal amplitude ratio between the nonideal state and ideal state with the carrier suppression ratio *M* is shown in Figure 9b. With the increase in *M*, the ratio gradually approaches 1, that is, it is also close to the ideal state.

**Figure 10:**
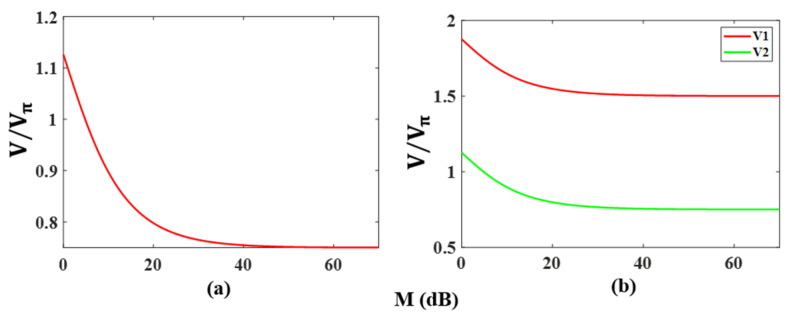
Variation in the amplitude of the electrical coding signal required to generate the phase-coded signal with the carrier rejection ratio. (**a**) in binary mode and (**b**) in quaternary mode.

Figure 10a shows the relationship between the change in voltage for π phase shifting in the binary phase-coded signal and the carrier rejection ratio *M*. As seen from Table 1, in an ideal situation, a binary phase-coded signal can be obtained when the amplitudes of both coding signals are $V=3V_{\pi }/4$. Since the carrier cannot be completely suppressed, it is necessary to increase the voltage difference V to offset the phase of π/2 in the additional term of Equation (19), to produce an accurate π phase shift. In the simulation, the amplitude of coding signal $S_{2}(t)$ is fixed, and only the amplitude of coding signal $S_{1}(t)$ is changed to obtain the desired phase offset. The results show that the larger the carrier residue is, the higher the required amplitude of the coding signal.

Similarly, the simulation results for the quaternary phase-coded signal are shown in Figure 10b. From Table 2, the quaternary phase-coded signal is generated based on the amplitude $V_{1}=3V_{\pi }/2$ of coding signal $S_{1}(t)$ and the amplitude $V_{2}=3V_{\pi }/4$ of coding signal $S_{2}(t)$. When the pattern of two coding signals is ‘1′, the generated phase shifts are π and π/2, respectively. Therefore, when the carrier is not suppressed well, it can also increase the amplitude $V_{1}$ or $V_{2}$ to obtain an accurate π or π/2 phase shift. Figure 10b shows the variation in the amplitude of coding signal $S_{1}(t)$ or $S_{2}(t)$ with the carrier suppression ratio M when only one coding signal amplitude is changed.

For phase-coded signals with a doubling frequency mode, similarly, the PDC-PM output signal can be expressed as

(21) $$E_{PDC-PM}(t)\approx \left\{\begin{array}{l} \left[A\exp(j\left[(\omega_{0}+\omega_{LO})t+\frac{\pi }{2}\right])+B\exp(j\omega_{0}t)\right]\exp(j\varphi_{1x}+j\varphi_{2x})\cdot \vec{e_{X}}\\ \left[C\exp(j\left[(\omega_{0}-\omega_{LO})t+\frac{\pi }{2}\right])+D\exp(j\omega_{0}t)\right]\exp(j\varphi_{1y}+j\varphi_{2y})\cdot \vec{e_{Y}} \end{array}\right\},$$

where *A* and *C* represent the amplitudes of the +1st-order sideband and −1st-order sideband of the DP-DPMZM output optical signal, respectively. *B* and *D* represent carrier residues in different polarization states.

The signals are injected into Pol at 45°, and then the electrical current after the PD can be expressed as

(22) $$i(t)\approx \left[\begin{array}{l} AC\cos(2\omega_{LO}t+\psi )-AB\sin(\omega_{LO}t)+CD\sin(\omega_{LO}t)\\ -AD\sin(\omega_{LO}t+\psi )+BC\sin(\omega_{LO}t+\psi ) \end{array}\right],$$

Equation (22) contains the desired doubling frequency phase-coded signal, as well as the fundamental frequency interference signal, in which the fundamental frequency signal only affects the spurious suppression ratio of the system. When the amplitudes satisfy A=C and B=D, the fundamental frequency terms can cancel each other, and a better spurious suppression ratio can be obtained.

In summary, the carrier suppression ratio has a greater influence on the amplitude of the coded signal in the fundamental frequency mode, but it has no impact in the doubling frequency mode. This can also be verified from the experimental results. In Figure 5 and Figure 6, the amplitude fluctuation of the phase-coded signal is obvious, while it is inapparent in Figure 7 and Figure 8. The simulation results show that when the carrier suppression ratio is higher than 20 dB, the phase shifting error of π/2 phase shifting is less than 5°. For 0, π/2, and π phase shifting, the amplitude fluctuation is less than 10%, 5‰, and 11.5%, respectively. Therefore, in fundamental frequency mode, it is necessary to select 90° HC with precision 90° phase shifting and use a high-precision bias control circuit with an automatic control function to ensure the acquisition of a high carrier suppression ratio CS-SSB signal. The phase shifting error can also be compensated for by increasing the low-frequency compensation voltage to the bias port of PDC-PM to obtain a high-precision phase-coded signal.

### 4.2. Influence of Polarization Crosstalk

Another factor that affects the experimental results is polarization crosstalk between sidebands, which is modeled after the definition of the polarization extinction ratio in [24], that is, the power ratio of a sideband optical signal in one polarization state to an optical signal of the same frequency in another polarization direction.

In the fundamental frequency mode, the output of the DP-DPMZM is

(23) $$E_{DP-DPMZM}(t)\approx \left[\begin{array}{c} A\left[\exp\left(j\left[(\omega_{0}+\omega_{LO})t+\frac{\pi }{2}\right]\right)+\alpha^{-1}\exp(j\omega_{0}t)\right]\cdot \vec{e_{X}}\\ C\left[\exp\left(j\omega_{0}t\right)+\varepsilon^{-1}\exp\left(j\left[(\omega_{0}+\omega_{LO})t+\frac{\pi }{2}\right]\right)\right]\cdot \vec{e_{Y}} \end{array}\right],$$

where α and ε are the polarization extinction ratios in the X and Y polarization directions, respectively, and α,ε≥1. The signal is input into PDC-PM for phase modulation, and PD is used for photoelectric detection.

(24) $$I(t)\approx \left[\begin{array}{l} \left(A^{2}\alpha^{-1}+C^{2}\varepsilon^{-1}\right)\cos\left(\omega_{LO}t+\frac{\pi }{2}\right)\\ +AC\alpha^{-1}\varepsilon^{-1}\cos\left(\omega_{LO}t+\frac{\pi }{2}-\psi \right)+AC\cos\left(\omega_{LO}t+\frac{\pi }{2}+\psi \right) \end{array}\right],$$

The output optical signal contains both a phase coded signal and an intensity coded signal due to polarization crosstalk. When α,ε=∞, the two polarization orthogonal signals tend to be ideal, and the PD output optical signal only contains the last term, thus obtaining an ideal phase-coded signal. The output expression can be simplified as

(25) $$I(t)\approx \mu \cos\left(\omega_{LO}t+\rho \right),$$

where $\mu =\sqrt{\xi^{2}+\chi^{2}}$ is the amplitude of the synthesized signal and $\rho =\arctan\left[\frac{\xi }{\chi }\right]$ is the phase of the synthesized signal, where $\xi =\left(A^{2}\alpha^{-1}+C^{2}\varepsilon^{-1}\right)+AC\left(1+\alpha^{-1}\varepsilon^{-1}\right)\cos\psi$ and $\chi =AC\left(\alpha^{-1}\varepsilon^{-1}-1\right)\sin\psi$.

**Figure 11:**
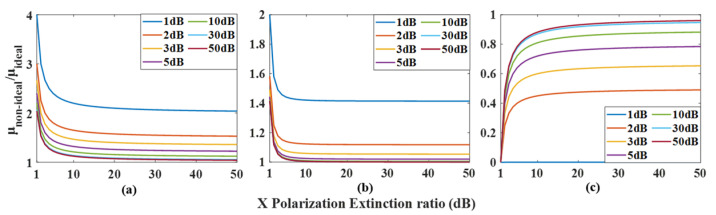
The variation in the microwave signal amplitude ratio between the nonideal state and ideal state with the polarization extinction ratio α at different polarization extinction ratio ε settings. (**a**) in ψ=0, (**b**) in ψ=π/2 and (**c**) in ψ=π.

**Figure 12:**
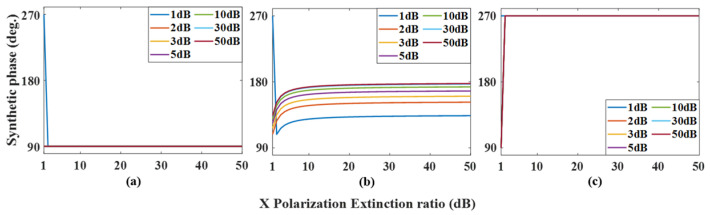
Relationship between the phase change of the output signal with the polarization extinction ratio α at different polarization extinction ratios ε in the fundamental frequency mode. (**a**) in ψ=0, (**b**) in ψ=π/2 and (**c**) in ψ=π.

In an ideal case, regarding the phase $\rho =\frac{\pi }{2}+\psi$ and amplitude μ=AC of the output optical signal, it can be clearly seen that ρ increases linearly with the increase of modulation voltage in PDC-PM, and the amplitude μ remains unchanged. When there is polarization crosstalk in the experiment, changing the phase modulation voltage of PDC-PM can make the phase shift ψ change correspondingly. As ψ=0, ψ=π/2, and ψ=π are often used in the experiment, the changes in the amplitude and phase of the synthesized signal with the polarization extinction ratio under three values are simulated, and the results are shown in Figure 11 and Figure 12, respectively. Figure 11a shows the variation in the amplitude ratio between nonideal and ideal cases with the extinction ratio α of X polarization when ψ=0. Different curves show different values of the extinction ratio ε of Y polarization, and it can be seen that with the increase in α and ε, the ratio gradually approaches 1. When the results of ψ=π/2 and ψ=π are shown in Figure 11b,c, it can be seen that when the values of α and ε are close to those of ordinary commercial modulators, that is, α,ε>30dB, the amplitude fluctuation is within the range of 6.5%. Figure 12 shows the changes in the output signal phase under different conditions. When ψ=0 and ψ=π, the synthesized phase meets the ideal situation, that is, $\rho =\frac{\pi }{2}+\psi$, while ψ=π/2, when α,ε>30dB, the error of the synthesized phase is less than 3.8°.

Similarly, in the doubling frequency mode, the output expression of the DP-DPMZM can be written as

(26) $$E_{DP-DPMZM}(t)=\left\{\begin{array}{l} A\left[\exp(j[(\omega_{0}+\omega_{LO})t+\frac{\pi }{2}])+\alpha^{-1}\exp(j[(\omega_{0}-\omega_{LO})t+\frac{\pi }{2}])\right]\cdot \vec{e_{X}}\\ C\left[\exp(j[(\omega_{0}-\omega_{LO})t+\frac{\pi }{2}])+\varepsilon^{-1}\exp(j[(\omega_{0}+\omega_{LO})t+\frac{\pi }{2}])\right]\cdot \vec{e_{Y}} \end{array}\right\},$$

At this time, the output photocurrent of the PD is

(27) $$I(t)\approx \left[\begin{array}{l} \left(A^{2}\alpha^{-1}+C^{2}\varepsilon^{-1}\right)\cos\left(2\omega_{LO}t\right)\\ +AC\alpha^{-1}\varepsilon^{-1}\cos\left(2\omega_{LO}t-\psi \right)+AC\cos\left(2\omega_{LO}t+\psi \right) \end{array}\right],$$

**Figure 13:**
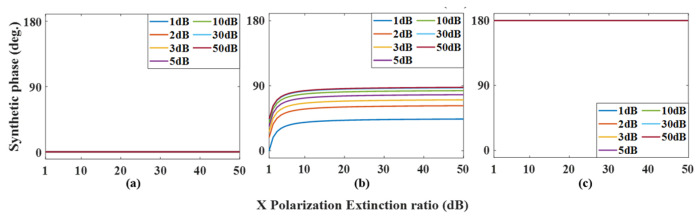
Relationship between the phase change of the output signal with the polarization extinction ratio α at different polarization extinction ratios ε in the doubling frequency mode. (**a**) in ψ=0, (**b**) in ψ=π/2 and (**c**) in ψ=π.

It also contains phase-coded signals and intensity-coded signals. In an ideal case, the phase of the output signal is ψ and the amplitude is AC. Simplifying the above formula can be expressed as

(28) $$I(t)\approx L\cos\left(2\omega_{LO}t+\gamma \right),$$

where the synthesized amplitude $L=\sqrt{\xi^{2}+{\left(-\chi \right)}^{2}}$ and the synthesized phase $\gamma =\arctan\left[-\frac{\chi }{\xi }\right]$. The amplitude of the synthesized signal is the same as that of the fundamental frequency mode; that is, when the polarization extinction ratio is in the commercial range, the synthesized amplitude is almost unaffected by polarization crosstalk and is close to the ideal state. Figure 13 simulates the change in the synthetic phase in the doubling frequency mode, and it can be seen that when the polarization extinction ratio reaches 30 dB, the phase shift error is less than 3.8°.

## 5. Conclusions

A photonics-assisted binary/quaternary phase-coded microwave signal generator with a fundamental/doubling carrier frequency has been proposed and demonstrated. The proposed doubling carrier frequency enables the generator to work in a frequency range well in excess of the frequency range of the LO source. An integrated waveguide PDC-PM has been designed and fabricated. The PDC-PM is driven by two independent two-level digital signals, and a binary or quaternary phase-coded signal can be realized by reasonably setting the amplitudes and sequence pattern. The two independent two-level digital signals can be directly generated through the IO interfaces of FPGA, instead of using high-speed AWG or other DAC systems to generate multilevel coding signals. In addition, the influence of residual carrier suppression and polarization crosstalk in non-ideal states on phase shifting has also been analyzed. Both binary and quaternary phase-coded microwave signals with frequencies of 5 GHz and 14 GHz in fundamental and doubling carrier frequency modes are generated. The experimental results show that it has good phase recovery accuracy and pulse compression performance. The proposed scheme features a compact configuration, large operational frequency range, and great tolerance to chromatic dispersion, which can be employed in high-frequency and wideband radar systems. However, it is still a challenge to solve the problems of carrier residual and polarization crosstalk. Therefore, in the future, we can try to design and fabricate an integrated device with a high polarization extinction ratio and modulation extinction ratio on thin film lithium niobate to avoid the impact of these problems as much as possible. In addition, the DP-DPMZM and PDC-PM used in the experiment were both manufactured based on lithium niobate material, which also provides the possibility of integrating them into a thin film of lithium niobate in the future.

## Figures and Tables

**Table 1:** The amplitudes and coding sequence pattern rules for binary phase-coded signals.

$b_{1}(t)$	$b_{2}(t)$	$V_{1}$	$V_{2}$	φ
0	0	$3V_{\pi ,PM}/4$	$3V_{\pi ,PM}/4$	0
1	1			π
0	0	$3V_{\pi ,PM}/2$	0	0
1	0			π

**Table 2:** The amplitudes and coding sequence pattern rules for quaternary phase-coded signals.

$b_{1}(t)$	$b_{2}(t)$	$V_{1}$	$V_{2}$	φ
0	0	$3V_{\pi ,PM}/2$	$3V_{\pi ,PM}/4$	0
0	1			π/2
1	0			π
1	1			3π/2

**Table 3:** Performance comparison of several photonics phase-coded microwave signal generators.

Ref.	Structure	Reconfigurable Carrier Frequency	Arbitrary Phase Coding	Suitable for Digital IO Interface	w/o * OBPF	w/o Electrical Phase Shifter	The Complexity of Control	Possibility of on-Chip Integration
[9]	serial	× **	√ **	No	w *	o		No
[10]	serial	×	√	No	o *	o		No
[11]	serial	√	×	No	o	o	normal	Yes
[12]	serial	√	√	No	o	w	special	No
[13]	serial	×	√	No	w	o	normal	No
[14]	serial	×	√	No	w	o	normal	No
[15]	serial	×	√	No	o	w	normal	No
[16]	parallel	×	√	No	w	o	normal	
[17]	parallel	√	×	No	o	w	special	
[18]	parallel	×	√	Yes	o	o	normal	
[19]	parallel	×	√	No	o	o	normal	
[20]	parallel	×	√	Yes	o	o	normal	
[21]	serial	×	√	No	o	w	special	No
The work	serial	√	√	Yes	o	o	normal	Yes

* w/o—with/without, w—with, o—without. ** ×/√—not having or having this function.

**Figure A1:**
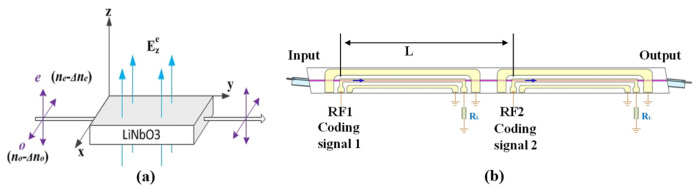
Structure and principle of the waveguide and electrode in PDC-PM. (**a**) The direction of electric and light field in chips, (**b**) structure of the waveguide and electrode in PDC-PM.

## Data Availability

Not applicable.

## Appendix A

The PDC-PM modulator is fabricated on a lithium niobate (LN) platform that adopts the Ti diffusion process. Ti diffusion only needs to occur in the waveguide part that conducts optical signals. Therefore, during the diffusion process, other parts need to be shielded. A Cr barrier layer is formed on the lithium niobate substrate by sputtering, and then the waveguide pattern is transferred to the surface of lithium niobate through processes such as bonding, lithography, development, etching, and so on. Ti diffusion operation is then achieved on the exposed surface of lithium niobate. Finally, the device is fabricated through annealing and electrodes.

In the modulation section of the waveguide, because lithium niobate is a uniaxial crystal, the linear electro-optical coefficients in different directions are different. The chip substrate is X-cut (along the crystal axis of the lithium niobate crystal), with light conducting in the Y direction and the modulated electric field mainly in the Z direction, as shown in Figure A1a.

Due to the Pockels effect of lithium niobate waveguides, changes in the refractive index of the waveguide can be achieved with an external electric field. In LN crystals, when an electric field is applied on the *z*-axis, as shown in Figure A1a, the electro-optic coefficients corresponding to extraordinary and ordinary are $\gamma_{33}$ (~30.8 pm/V) and $\gamma_{13}$ (~8.6 pm/V), respectively. Therefore, the refractive indices corresponding to extraordinary and ordinary are as follows

(A1) $$\left\{\begin{array}{c} n_{x}=n_{o}-\Delta n_{o},\;\;\;\Delta n_{o}=\frac{1}{2}\gamma_{13}n_{o}^{3}E_{z}^{e}\\ n_{z}=n_{e}-\Delta n_{e},\;\;\;\Delta n_{e}=\frac{1}{2}\gamma_{33}n_{e}^{3}E_{z}^{e} \end{array}\right.$$

where $n_{e}$ and $n_{o}$ are the refractive index of extraordinary and ordinary waves, respectively, and $E_{z}^{e}$ is the intensity of the electric field applied in the *z*-axis direction.

When the polarization multiplexed optical signal is input into the Ti diffusion waveguide, the X polarization state corresponds to the extraordinary wave, and the Y polarization state corresponds to the ordinary wave. While applying a voltage to the waveguide through electrodes, the changes in the waveguide refractive index, which result in a phase change of the two polarized optical signals can be written as

(A2) $$\left\{\begin{array}{c} \varphi_{x}=\frac{2\pi }{\lambda }\Delta n_{e}L\\ \varphi_{y}=\frac{2\pi }{\lambda }\Delta n_{o}L \end{array}\right.$$

where λ is the wavelength, and L is the electrode length.

Therefore, when the polarization multiplexed optical signal is modulated by an external electric field through a Ti diffusion phase modulator, which will introduce an optical phase $\varphi_{y}$ and $\varphi_{x}$ to each of the two orthogonally polarized signals, and the optical phase difference $\left(\varphi_{x}-\varphi_{y}\right)$ is linearly related to the voltage of the PDC-PM. This is followed by a 45° polarizer, and the optical phase difference at the input of the PD is given by

(A3) $$\varphi_{x}-\varphi_{y}=\frac{2\pi }{\lambda }(\Delta n_{e}-\Delta n_{o})L$$

With the production process, electrode parameters, and the overlap integral of the light field passing through the electrode area and electric field, and so on, the modulation efficiency of X polarization is approximately 3 times that of Y polarization. Figure A1b shows the structure of a PDC-PM, which includes two polarization-dependent phase modulators, each of which contains an RF port.
